# Response probabilities and response-mode preferences in a self-administered survey

**DOI:** 10.1186/s13104-019-4328-7

**Published:** 2019-05-27

**Authors:** Ingeborg Strømseng Sjetne, Hilde Hestad Iversen, Olaf Holmboe, Jon Helgeland

**Affiliations:** Health Services Research, Norwegian Institute of Public Health, Oslo, Norway

**Keywords:** Data collection, Health-care surveys, Response rate, Response mode, Response-homogeneity groups, Patient-reported experiences measures, Survey, Maternity care

## Abstract

**Objective:**

Response rates in surveys continue to fall, and electronic online versions are increasingly replacing paper questionnaires in order to save costs and time. This can influence the composition of the respondent group in surveys. Using data from a national survey of patient experiences with maternity care, we aimed to (1) classify all of the women invited to participate in the study according to their different probabilities of responding, based on registry data, and (2) classify all of the respondents according to different probabilities of choosing a paper questionnaire when an online alternative was available, based on registry and self-reported data.

**Results:**

We found that the likelihood of responding to surveys is strongly influenced by background variables, with the age, number of previous births and geographic origin predicting the response probability (range 0.25–0.73). Education level predicted the likelihood of choosing a paper questionnaire. Women with less education would more likely (probability 0.50) than women with more education (probability 0.38) choose a paper questionnaire rather than answering online.

## Introduction

A high response rate is a common goal when conducting surveys, but this has generally been declining for decades, and there is little hope that it will change for the better [1]. Technological developments make electronic online versions increasingly available, and replacing the use of paper questionnaires with digital solutions will save costs and time.

One approach to understanding the effect of non-responses is to investigate how the backgrounds of subjects influence their propensity to respond. The relevant variables may be available from registries or from a survey itself.

After issuing a white paper in 2009 about pregnancy, birth and postnatal care, the Norwegian Ministry of Health and Care Services commissioned a national survey among the users of the relevant health-care services. All phases of the care were to be included, with special attention paid to immigrant women. The Norwegian Institute of Public Health is responsible for conducting surveys to collect patient-reported experience measures among health-care users.

The aims of this paper are to present the following observations that were a side product in the national survey of patient-reported experiences with maternity care in Norway in 2011:

1. To classify all women invited to participate in the study according to their different probabilities of responding, based on individual data collected from registries.
2. To classify all of the respondents according to their different probabilities of using a paper questionnaire when an online version is available as an alternative, based on individual data collected from registries and additional respondent-reported data.

## Main text

### Methods

#### A national survey

A questionnaire and data collection routines were developed for this specific population. The final questionnaire consisted of 145 items in total (comprising 16 pages in the printed version) collecting the women’s description of their experiences and sociodemographic information [2].

We included women who gave birth in a birthing institution or hospital department during the last quarter of 2011 and were aged 16 years or older. Based on our experiences when performing previous patient surveys, the sample size was set to 400 potential respondents in each institution. All women at hospitals with less than 400 births during the inclusion period were included, while a random sample of women was drawn from hospitals with more than 400 births. The Medical Birth Registry, which also provided clinical information about the women, performed the sampling. Statistics Norway provided data about the countries where the women were born, and this information was coded in four categories: (1) Norway; (2) Asia, Turkey, Africa, South America; (3) Eastern Europe; or (4) Western Europe, North America, Oceania.

Before the national study, the postal and electronic alternative data collection modes were studied in a randomized comparison of effectiveness and costs [3]. Based on the findings in this study, all the included women were contacted by mail in the national survey about 17 weeks after the birth. The initial invitation offered an electronic response option only, and a printed questionnaire was enclosed in both of two reminders that were subsequently sent to non-respondents.

#### Statistical procedures

The Response Homogeneity Group (RHG) model was used to reduce bias from nonresponse [4] and to model response preference. In this model, the initial sample is partitioned into groups based on data in the sampling frame or registry. The response probability is assumed constant within each group, and is estimated from the observed response rates.

In addition to being an important step in weighting procedures, the models produce observations about the composition of the survey sample that are valuable per se.

To identify predictors for responding, we initially tested 15 variables that we hypothesized to be associated with responding, and that were available in our data set. The candidate variables were tested in bivariate logistic regression models with response as the outcome variable. The woman’s age, number of previous births, geographic origin (four categories), Caesarean section and episiotomy were significantly associated with response to the survey (*p* < 0.001). These variables were all entered into a multivariate regression model for response probability, addressing the first aim of the study. We used the recursive partitioning method with bootstrapping to construct a regression tree [5–7], using the rpart package in R, version 3.0.3 [8].

In order to classify the participating women according to their probability of responding via a printed questionnaire when there was the alternative of answering online (to address the second study aim), we selected potential predictors in the same way as described above and supplemented with self-reported data from the respondents. The variables included were the women’s age, number of previous births, region of birth, Caesarean section, instrument use, episiotomy, size of the municipality of residence, self-reported employment status, self-rated health and education level.

The register data were complete, and the item missing rates were all below 2.4% in the self-reported variables.

For both models, we set the minimum size of groups to 100 women per RHG, to avoid generating RHGs with very few women.

### Results

Of the 8670 sampled women, 4904 (56.6%) responded. Table 1 lists the characteristics of the groups of women with the same probability to respond, RHGs. The response probability in the eight RHGs varied from 0.25 to 0.73 (Table 1).

**Table 1:** Response homogeneity groups

RHG	Region of birth	Age (years)	Previous births	Group size	Response probability
1	Asia, Turkey, Africa, South America or Eastern Europe	Any	≥ 2	392	0.25
2	Asia, Turkey, Africa, or South America	≤ 28	0 or 1	351	0.29
3	Eastern Europe	≤ 28	0 or 1	288	0.47
4	Asia, Turkey, Africa, South America or Eastern Europe	≥ 29	0 or 1	642	0.49
5	Norway, Western Europe, North America or Oceania	≤ 24	Any	1268	0.50
6	Norway, Western Europe, North America or Oceania	≥ 25	≥ 2	1485	0.53
7	Norway, Western Europe, North America or Oceania	≥ 25	1	2165	0.61
8	Norway, Western Europe, North America or Oceania	≥ 25	0	2079	0.73

Table 2 shows the results of applying the same modelling procedure to predicting whether the respondents chose to respond on a paper questionnaire. Only educational level was eventually retained in the model.

**Table 2:** Response homogeneity groups for the use of a paper questionnaire among survey respondents

RHG	Education level	Group size	Probability of using a paper questionnaire
1	Primary school to high school	1630	0.50
2	College or university undergraduate to postgraduate	3274	0.38

### Discussion

In this side product to a national survey, we have confirmed that the likelihood of responding to surveys is strongly influenced by background characteristics. The response probability varied considerably (from 0.25 to 0.73) among groups in our sample. The age, number of previous births and geographic origin predicted the response probability, and education level alone predicted the probability of respondents opting to use a paper questionnaire as a response mode.

To our knowledge, there are no previous publications about using this specific approach to explore survey participation in different sample subgroups. That survey participation in general may vary between groups is a known phenomenon in surveys using self-administered data collection. In a similar national survey of experiences with maternity care in the United Kingdom in 2010, the respondents were more likely to be older, to be married, to be living in the least deprived areas and to be born in the United Kingdom, compared to non-respondents [9]. Our analysis also showed that response probability was larger for older women and women from Norway or other western countries. In Norway, the immigrant population has increased markedly in Norway over the past 20 years, from approximately 5% in 1999 to 17% in 2018 [10]. Most likely, this has consequences for the response rates in many populations.

A review of studies comparing response rates between different data collection methods, found that response rates in web surveys are lower than in alternative response modes, but that web surveys are the most efficient with regard to time and costs [11]. Internet use in Norway in 2012 was ubiquitous among women of child-bearing age, with 100% of that population having used the Internet within the previous 3 months, and digital skills among the general Norwegian population are among the best in Europe [12, 13]. We therefore assume that online responding is an easily accessible option in this relatively young population. In a study comparing postal versus mixed mode (internet and paper questionnaire in combination) the authors concluded that a mixed mode solution should be a method to consider, in particular if the target population is young and well educated [14]. In showing that education level predicted response mode preference, our study draws attention to possible consequences of ceasing to offer a postal response mode in populations that also include older persons. According to Norwegian statistics, 49% of the general population between 30 and 34 years was educated at college or university level in 2017, compared to 22% among persons older than 66 years [15, 16]. Thus, there is a risk that older persons will be underrepresented in surveys that offer online responding only.

The availability of a large high-quality data set provided the main motivation for reporting on this side product. We believe that our findings show that exploring the consequences of population diversity is highly relevant, and that the findings represent helpful input in informing considerations before deciding on future data collection procedures.

## Limitations

The present data were collected in 2012, which could be regarded as a limitation given the rapid ongoing developments in this field. We believe that even if response rates continue to decrease, it can be assumed that patterns like those we found are still present, and hence worthy of attention.

Future studies should include a larger set of background data about the complete population, such as the education level of non-respondents.

## Data Availability

The data from the current study are available to named researchers at the Norwegian Institute of Public Health.
